# Apathy in Parkinson’s Disease: A Retrospective Study of Its Prevalence and Relationship With Mood, Anxiety, and Cognitive Function

**DOI:** 10.3389/fpsyg.2021.749624

**Published:** 2021-11-22

**Authors:** Jennifer A. Foley, Lisa Cipolotti

**Affiliations:** ^1^Department of Neuropsychology, National Hospital for Neurology and Neurosurgery, London, United Kingdom; ^2^UCL Queen Square Institute of Neurology, London, United Kingdom

**Keywords:** apathy, Parkinson’s disease, depression, anxiety, neuropsychology

## Abstract

Apathy is thought to be an important clinical feature of Parkinson’s disease (PD). However, its prevalence ranges greatly across studies because of differing definitions, assessment tools, and patient inclusion criteria. Furthermore, it remains unclear how the presentation of apathy in PD is related to mood disorder and/or cognitive impairment. This study sought to examine the prevalence of a pure apathy syndrome in PD, distinct from both depression and anxiety, and reveal its associated cognitive profile. A retrospective study was performed on 177 PD patients who had completed measures of apathy [Apathy Evaluation Scale (AES)] and mood functioning [Hospital Anxiety and Depression Scale (HADS)] and had undergone extensive neuropsychological assessment, using measures of intellectual functioning, memory, executive function, attention, language, visual processing, and cognitive speed; 14.7% of the sample indicated clinically significant levels of apathy, but this nearly always co-presented with depression and/or anxiety, with cases of “pure” apathy very rare (2.8%). On extensive cognitive assessment, patients with mood disorder performed worse on a measure of non-verbal intellectual functioning, but patients with additional apathy or apathy only demonstrated no further losses. The syndrome of apathy in PD greatly overlaps with that of depression and anxiety, suggesting that apathy in PD may be in large an epiphenomenon of mood disorder, with no specific neuropsychological features.

## Introduction

Apathy is thought to be a non-motor symptom of Parkinson’s disease (PD). It severely affects daily function (Isella et al., 2002; Pedersen et al., 2010; Leroi et al., 2012) and quality of life (Benito-León et al., 2012) and is a harbinger of dementia (Dujardin et al., 2009). It increases carer burden (Leiknes et al., 2010; Leroi et al., 2012) and has been rated by carers as the most distressing symptom of the condition (Leiknes et al., 2010). However, despite awareness of its important clinical impact, we still know very little about its true prevalence or relationship with mood disorder and/or cognitive impairment.

Prevalence rates remain greatly debated, with figures ranging from 12% to 70% across different studies and even differing significantly across two extant meta-analyses (den Brok et al., 2015; Mele et al., 2019). This variation is caused by several important factors, including differing conceptualisations, assessment tools, and patient inclusion criteria.

First, the definition of apathy has changed significantly over time. Initially meaning a state of mind undisturbed by the passions, modern definitions now describe apathy as a disabling loss of feeling, emotion, interest, or concern (Levy and Dubois, 2005). Marin (1991, 1996) emphasised a reduction in motivation, not explained by a diminished level of consciousness, cognitive impairment, or emotional distress, and this was later operationalised as reductions in goal-directed behaviour and cognition (Starkstein, 2000; van Reekum et al., 2005). Others have argued that this definition is insufficiently objective and emphasised the need for a measureable and quantitative reduction in self-generated voluntary and purposeful behaviour (Levy and Dubois, 2005). Others still have emphasised the affective disorder and defined apathy as a loss of emotional feeling and receptivity (Sims, 2003), overlapping with the notion of anhedonia. Current (proposed) diagnostic criteria attempt to draw together these differing conceptualisations and define apathy as a reduction in motivation from a previous level, present for at least 4 weeks, associated with diminished goal-directed behaviour, cognitive activity, and/or emotion, and causing functional impairments (Robert et al., 2009). However, despite these proposals, there remains no universally-agreed standard diagnostic criteria and apathy as a syndrome remains absent from current diagnostic manuals.

Second, the historical lack of consistency in the definition of apathy has led to difficulties in developing validated assessment scales. A review commissioned by the Movement Disorders Society noted that although several measures of apathy are available, external validation has not yet been possible because of the absence of any gold standard (Leentjens et al., 2008). As a consequence, existing scales may vary in their focus, reflecting authors’ theoretical stance and limiting correlation between measures. Scales also vary in how they are scored; self- or informant-ratings, or clinician interview. Although some studies have reported correlations between these (Pluck and Brown, 2002), any assessment of apathy is open to bias (Schiehser et al., 2013) and despite initial concerns that self-ratings may be artificially deflated by insufficient insight or denial (Marin, 1991), recent studies have shown that self-ratings of apathy are actually higher those from proxies (Radakovic et al., 2018; Valentino et al., 2018). These findings may suggest that self-ratings are the most sensitive for the detection of apathy in PD.

Lastly, apathy rates also vary according to differing inclusion criteria, with some studies excluding patients with comorbid mood disorder. Although some have argued that apathy is simply an epiphenomenon of low mood (Bogart, 2011), with significant overlap between features of both (Gallagher and Schrag, 2012), particularly in the feature of anhedonia, several studies have argued that the two states are dissociable (Levy et al., 1998; Isella et al., 2002; Pluck and Brown, 2002; Aarsland et al., 2007; Santangelo et al., 2009; Kirsch-Darrow et al., 2011; Cubo et al., 2012) and that excluding patients with comorbid depression reveals the prevalence of a “pure” apathy syndrome in PD. However, these studies have often failed to consider the presence of comorbid anxiety, which has also been shown to be associated with both depression and apathy in PD (Aarsland et al., 1999, 2007; Kulisevsky et al., 2008; Starkstein and Brockman, 2011; Maillet et al., 2016). Overlap in apathy and anxiety may reflect not only shared diagnostic features, particularly of activity avoidance, but also the consequences of dopaminergic and serotonergic disruption in the genesis of neuropsychiatric symptoms in PD (Maillet et al., 2016). This means that the true rate of pure apathy as a syndrome entirely separate from both depression and anxiety in PD remains unknown.

Existing studies have also differed according to whether or not they have excluded patients with dementia (Starkstein et al., 1992; Santangelo et al., 2015). Previous studies have suggested that apathy in PD is associated with cognitive decline (Dujardin et al., 2009), and therefore it may not be appropriate to exclude such patients, but rather more prudent to consider the relationship between apathy and general global cognitive functioning.

Notwithstanding these differences, there have been several attempts at elucidating the relationship between apathy and cognitive function. A few studies have reported lower performance on tests of memory (Starkstein et al., 1992; Dujardin et al., 2009) and visual processing (Santangelo et al., 2015; Alzahrani et al., 2016). Several studies have reported lower performance on measures of executive function, most commonly on measures of verbal generation, such as letter and category fluency (Starkstein et al., 1992; Pluck and Brown, 2002; Zgaljardic et al., 2007; Dujardin et al., 2009; Pedersen et al., 2010), and verbal inhibition, as measured by the Stroop (Pluck and Brown, 2002; Pedersen et al., 2010). However, the conclusions that can be drawn from these studies are limited by their diverse assessment methods, with some studies relying upon clinician ratings of apathy only (Alzahrani et al., 2016) and/or a minimal neuropsychological test battery, using screening tools only (Dujardin et al., 2014). Most have also failed to examine the impact that depression may have upon the neuropsychological profile, including patients with both apathy and depression (Pluck and Brown, 2002), and even fewer have considered the role of anxiety. Several studies have also failed to include any measure of general cognitive function (Zgaljardic et al., 2007), limiting interpretation of the specific nature of any cognitive correlates of apathy.

Therefore, the aims of the current study were to provide an improved understanding of:

(1) How apathy interrelates with both depression and anxiety, in order to determine its prevalence as an entirely independent and pure syndrome in PD;
(2) The cognitive profile of apathy in PD, as assessed using extensive neuropsychological assessment, including measures of general cognitive function, memory, visual processing, and executive function.

## Materials and Methods

### Participants

Patients with a diagnosis of idiopathic PD, according to Queen Square Brain Bank criteria, who attended the Neuropsychology Department of the National Hospital for Neurology and Neurosurgery (Queen Square, London, United Kingdom), were retrospectively evaluated for eligibility. The following exclusion criteria were employed: (i) concomitant or previous history of neurologic, traumatic, psychiatric, or systemic disorder; (ii) a history of alcohol and/or drug abuse; (iii) not being a native English speaker. A total of 177 patients (117 male, 60 female) met the inclusion criteria for the study.

### Apathy and Mood Assessment

Apathy was assessed using the 18-item self-rated Apathy Evaluation Scale (AES; Marin et al., 1991). In the absence of a gold standard measure, this scale was chosen because of its superior psychometric properties: it has good reliability (Marin et al., 1991) and validity (Pluck and Brown, 2002; Santangelo et al., 2015), with high sensitivity and specificity for detecting apathy in PD (both 0.9; Santangelo et al., 2015; Mele et al., 2019). Each item is rated on a four-point Likert-type scale, with a maximum total score of 72 and scores of ≥ 38 indicating clinically significant apathy. Mood was assessed using the self-rated Hospital Anxiety and Depression Scale (HADS; Zigmond and Snaith, 1983) with each subscale having a total score of up to 21 and a score of ≥ 8 on either indicating clinically significance.

### Cognitive Assessment

All of the participants completed a battery of standardised neuropsychological assessments, which included a cognitive screen and measures of premorbid level of intellectual functioning, current level of intellectual functioning, memory, executive function, attention, language functioning, visual processing, and speed of information processing. All scores were converted into scaled scores, where appropriate. Cognitive function was screened using the Mattis Dementia Rating Scale, Second Edition (Jurica et al., 2001), with a maximum score of 144. Premorbid level of intellectual functioning was estimated using the National Adult Reading Test (NART; Nelson, 1982) to predict full-scale IQ (PFSIQ). Current level of intellectual functioning was assessed using tasks from the Wechsler Adult Intelligence Scale – Third Edition (WAIS-III; Wechsler, 1997). Verbal IQ (VIQ) was pro-rated using the Vocabulary, Similarities, Arithmetic, and Digit Span subtests. Performance IQ (PIQ) was pro-rated using the Picture Completion and Matrix Reasoning subtests. Verbal and PIQ scores were combined to generate a measure of full-scale IQ (FSIQ). Memory for recognition and recall was assessed. Recognition memory was assessed using the Words and Faces Recognition Memory Tests (RMT; Warrington, 1984). These tests have a maximum score of 50. Recall memory was assessed using the Shapes and People subtests from the Doors and People (D&P) Test (Baddeley et al., 1994) and calculated as scaled scores. Executive functioning was assessed using measures of phonemic fluency, the Stroop (Trenerry et al., 1989) and the Hayling Sentence Completion Test (Burgess and Shallice, 1997). Phonemic fluency score was the total number of correct words starting with “S” generated in 60 s. Stroop score was the total number of correct responses generated in 2 min, with a maximum of 112. Hayling Sentence Completion Test score was calculated as a scaled score. Attention was assessed using the Elevator Counting subtest from the Test of Everyday Attention (Robertson et al., 1994), with a maximum score of 7, and the Digit Span subtest scaled score from the WAIS-III (Wechsler, 1997). Language functioning was assessed using the Graded Naming Test, with a maximum score of 30 (GNT; McKenna and Warrington, 1983) and the Vocabulary subtest scaled score from the WAIS-III (Wechsler, 1997). Visual processing was assessed using the Silhouettes and Cube Analysis subtests from the Visual Object and Space Perception Battery, with a maximum score of 30 and 20, respectively (VOSP; Warrington and James, 1991). Speed of information processing was assessed using the Symbol Search (SS) and Digit Symbol Coding (DSC) subtest scaled scores from the WAIS-III (Wechsler, 1997).

The research was done in accordance with the Helsinki declaration and the Institute of Neurology Joint Research Ethics Committee UCLH, NHS Trust Research and Development Directorate.

### Statistical Analysis

Where possible, raw scores were transformed into scaled scores, with reference to the available normative data described in the manuals for each of the measures listed. Mean and standard deviations were calculated for each of the variables. Normality of distribution was assessed using the Kolmogorov–Smirnov test and, if significant, by examining the *z*-scores for skewness and kurtosis. Homogeneity of variance was assessed using Levene’s test. Unless otherwise stated, all data met the assumptions of normality and homogeneity of variance. Data were analysed using Pearson correlation analyses to explore associations between self-ratings on the apathy and mood scales, and cognitive function. The sample was split into two apathy subgroups according to apathy median score, and subject to χ*^2^* analyses to explore associations between level of apathy and frequency of mood disorder, and *t*-tests to elicit any significant subgroup differences. The sample was also split into four subgroups according to the presence or absence of apathy and/or mood disorder, and subject to analyses of variance to compare subgroup differences in self-ratings of apathy and mood, background demographic and disease variables, and neuropsychology test scores. *Post hoc* analyses were adjusted for multiple comparisons using Bonferroni correction. Finally, a backward multiple regression was used to reveal significant predictors of self-ratings of apathy. All analyses were conducted using IBM SPSS Statistics Data Editor version 24.

## Results

### Apathy and Mood

In the 177 PD patients, AES apathy scores ranged from 18 to 75, with an overall mean of 28.95 (SD = 8.88). A total of 26 (14.7%) indicated clinically significant levels of apathy (score of ≥ 38) and nearly half (49.7%) indicated mood disorder.

There was a high overlap between apathy and mood disorder. Of the 26 patients with apathy, 21 (80.8%) also indicated comorbid mood disorder (anxiety: *n* = 4; depression: *n* = 4; anxiety and depression: *n* = 13). Of the remaining 153 patients who did not report apathy, 67 endorsed mood disorder (anxiety: *n* = 40; depression: *n* = 11; anxiety and depression: *n* = 16) and 84 patients did not. Apathy was significantly associated with mood disorder, with high correlations between self-ratings of apathy and both anxiety (*r* = 0.42, *p* < 0.001) and depression (*r* = 0.61, *p* < 0.001). Median split analyses confirmed that self-ratings of apathy were significantly higher in those endorsing high levels of anxiety [*t*(170.90) = −4.18, *p* < 0.001] and depression [*t*(136.27) = −7.41, *p* < 0.001], and that those with high self-ratings of apathy were more likely to have clinically significant levels of anxiety [χ^2^(1) = 9.80, *p* < 0.01] and depression [χ^2^(1) = 22.57, *p* < 0.001].

Parkinson’s disease patients were then grouped according to whether they indicated (1) pure apathy, *n* = 5; (2) apathy and mood disorder, *n* = 21; (3) pure mood disorder, *n* = 67; or (4) neither apathy nor mood disorder (*n* = 84), as shown in Table 1. There were no significant differences in age, NART PFSIQ, gender, disease duration, levodopa equivalent dosage, or DRS-2 scores between these subgroups, as shown in Table 2.

**TABLE 1:** PD patients’ self-ratings of apathy, anxiety, and depression [mean (SD)].

	*PD* + *pure apathy*	*PD* + *apathy* + *mood disorder*	*PD* + *pure mood disorder*	*PD no apathy or mood disorder*
	(*n* = *5*)	(*n* = *21*)	(*n* = *67*)	(*n* = *84*)
AES Apathy	44.20 (7.63)	46.14 (9.27)	27.69 (4.58)	24.75 (4.37)
HADS Anxiety	5.40 (1.67)	11.43 (4.52)	9.75 (3.02)	4.63 (1.94)
HADS Depression	5.60 (0.55)	11.05 (4.11)	6.54 (2.90)	3.45 (1.96)

*AES, Apathy Evaluation Scale; HADS, Hospital Anxiety and Depression Scale.*

**TABLE 2:** Demographics of PD patient groups [mean (SD)].

	*PD* + *pure apathy*	*PD* + *apathy* + *mood disorder*	*PD* + *pure mood disorder*	*PD no apathy or mood disorder*
	(*n* = *5*)	(*n* = *21*)	(*n* = *67*)	(*n* = *84*)
Age (years)	67.00 (4.00)	59.67 (10.69)	60.75 (8.65)	60.85 (8.23)
NART PFSIQ	113.60 (10.09)	108.76 (10.46)	108.30 (12.42)	110.03 (12.15)
Gender (% male)	80.0%	56.7%	56.7%	73.8%
Disease duration (years)	11.60 (3.21)	9.57 (5.80)	12.16 (5.81)	11.11 (4.61)
Levodopa equivalent dosage (mg)	847.80 (296.27)	976.62 (605.09)	1125.70 (575.12)	1269.96 (627.28)
DRS-2	136.25 (10.97)	139.93 (3.67)	138.50 (5.46)	139.86 (4.46)

*NART PFSIQ, National Adult Reading Test Premorbid Full-Scale IQ; DRS-2, Dementia Rating Scale-2.*

### Cognitive Performance

As shown in Table 3, there were significant group differences in PIQ [*F*(3,173) = 4.04, *p* < 0.01; *r* = 0.26], FSIQ [*F*(3,173) = 3.00, *p* < 0.05; *r* = 0.22], phonemic fluency [*F*(3,173) = 3.19, *p* < 0.05; *r* = 0.25], and Digit Symbol scores [*F*(3,156) = 3.01, *p* < 0.05; *r* = 0.23]. *Post hoc* comparisons adjusted for multiple comparisons revealed that PIQ scores were significantly lower in PD patients with pure mood disorder than PD patients without apathy or mood disorder (*p* < 0.0125). Initial analyses also revealed that FSIQ was lower in PD patients with pure mood disorder than PD patients without apathy or mood disorder, but this was no longer significant after adjustment for multiple comparisons. *Post hoc* comparisons revealed that phonemic fluency was lower in PD patients with pure apathy than both the PD patients with mood disorder and PD patients without apathy or mood disorder, but these findings were no longer significant after adjustment for multiple comparisons. *Post hoc* comparisons revealed no significant group differences in Digit Symbol scores.

**TABLE 3:** PD patient groups’ performance on cognitive assessment [mean (SD)].

	*Test (median score*)	*PD* + *pure apathy*	*PD* + *apathy* + *mood disorder*	*PD* + *pure mood disorder*	*PD no apathy or mood disorder*
		(*n* = *5*)	(*n* = *21*)	(*n* = *67*)	(*n* = *84*)
*IQ*	VIQ (105)	100.20 (13.48)	105.48 (15.96)	102.52 (14.09)	106.75 (13.23)
	PIQ (99)	87.60 (15.08)	99.90 (20.25)	94.94 (15.43)*	103.25 (16.24)
	FSIQ (103)	94.00 (12.31)	103.14 (17.31)	99.22 (14.16)	105.54 (13.87)
*Memory*	RMT Words (47)	43.75 (7.76)	44.58 (6.61)	46.09 (5.66)	46.70 (3.85)
	RMT Faces (39)	39.50 (5.51)	41.47 (5.77)	39.07 (6.36)	41.26 (5.45)
	D&P People SS (9)	6.50 (1.73)	9.25 (3.47)	9.26 (3.07)	9.63 (3.05)
	D&P Shapes SS (11)	9.33 (2.89)	10.27 (2.60)	10.07 (2.65)	10.15 (2.63)
*Executive*	‘S’ fluency (17)	9.00 (5.24)	15.93 (6.75)	17.37 (5.10)	17.64 (6.92)
	Stroop (82)	79.33 (13.43)	77.00 (23.68)	79.69 (24.54)	82.63 (22.77)
	Hayling SS (6)	4.25 (1.71)	4.76 (1.81)	5.24 (1.46)	5.35 (1.38)
*Attention*	Elevator Counting (7)	6.75 (0.50)	6.42 (1.22)	6.75 (0.55)	6.61 (0.99)
	Digit Span SS (10)	9.25 (1.71)	10.71 (3.10)	10.41 (2.88)	10.21 (2.55)
*Language*	GNT (23)	21.40 (3.91)	23.38 (3.53)	21.66 (3.70)	22.88 (4.40)
	Vocabulary SS (12)	12.00 (3.54)	11.59 (2.62)	11.37 (2.86)	12.30 (2.95)
*Visual*	Silhouettes (21)	18.00 (2.16)	21.10 (4.23)	20.63 (4.27)	21.59 (3.75)
*Speed*	Digit Symbol (47)	42.00 (9.85)	41.65 (12.18)	43.95 (16.28)	50.38 (15.03)
	Symbol Search (25)	19.33 (3.06)	21.90 (6.91)	27.43 (10.64)	25.07 (7.83)

*p < 0.05 compared to PD no apathy or mood disorder; VIQ, PIQ, FSIQ, Wechsler Adult Intelligence Scale – Third Edition, Verbal IQ, Performance IQ, Full-Scale IQ; RMT, Recognition Memory Test; D&P, Doors & People; GNT, Graded Naming Test; Hayling SS, Hayling Scaled Score; Digit Span SS, Wechsler Adult Intelligence Scale – Third Edition, Digit Span Scaled Score; Vocabulary SS, Wechsler Adult Intelligence Scale – Third Edition.

### Relationship Between Apathy, Mood, and Cognitive Function

Pearson correlational analyses were conducted to determine the relationship between apathy, mood, and PIQ. As shown in Table 4, self-ratings of apathy were significantly related to self-ratings of anxiety and depression, but not PIQ. In order to reveal which of these contributed most to self-ratings of apathy, a backward multiple regression was conducted. This revealed that HADS anxiety and depression scores, but not PIQ, were significant predictors of self-rated apathy, accounting for 37.6% of the variance in AES scores [*F*(2,173) = 52.08, *p* < 0.001]. As expected, self-ratings of anxiety and depression correlated, but no significant multicollinearity (all *r* < 0.9; tolerance = 0.687). Of these, depression appeared to be the most predictive (ß = 1.32, *p* < 0.001).

**TABLE 4:** Correlation matrix of apathy, mood, and FSIQ.

	*AES Apathy*	*HADS Anxiety*	*HADS Depression*
*HADS Anxiety*	0.42**		
*HADS Depression*	0.61**	0.56**	
*PIQ*	−0.07	−0.21**	−0.13

**p < 0.001. HADS, Hospital Anxiety and Depression Scale; PIQ, Performance IQ.

## Discussion

The first aim of this study was to reveal the prevalence of apathy in PD. Initial assessment of self-rated apathy in our sample of 177 patients revealed a prevalence rate of 14.7%. This rate is at the lower end of previous estimates (den Brok et al., 2015; Mele et al., 2019). Variation in previous prevalence figures has been in part caused by significant diversity in approaches to assessment. Whereas many previous studies have chosen to examine clinician or informant ratings of apathy, recent studies have suggested that self-ratings are superior for detecting apathy in PD (Radakovic et al., 2018; Valentino et al., 2018). Although there are numerous measures of self-rated apathy available, few have been designed for PD. Therefore, in this study, we assessed self-ratings of apathy using a tool that has been shown to be reliable and valid for detecting apathy, with high sensitivity and specificity for this population.

Variation in prevalence figures has also been caused by differing inclusion and exclusion criteria, with some excluding patients with comorbid mood disorder. Moreover, studies that have examined the relationship between apathy and mood have tended to focus on depression only, failing to consider the impact of anxiety. Therefore, it remains unclear how the presentation of apathy in PD is related to attendant mood disorder. When we excluded those who also endorsed depression and/or apathy, only five patients (2.8%) indicated clinically significant levels of apathy that were entirely independent of mood disorder. Moreover, those with higher levels of depression or anxiety were significantly more likely to endorse apathy. These findings are consistent with previous reports of a high concomitant rate between apathy and depression (Cubo et al., 2012), and extend these findings to include anxiety as well.

It has been noted previously that depression correlates with self-ratings of apathy, but not with informant ratings (Skidmore et al., 2013). This suggests that the subjective experience of apathy may be in part a mood-based phenomenon. Certainly, there is considerable overlap between apathy and depression, with both featuring a reduction in self-generated voluntary and purposeful behaviour, and some previous definitions of apathy emphasising the affective disorder (Sims, 2003). Thus far the overlap between apathy and anxiety has largely been ignored but may be explained in part by a shared feature of activity avoidance. Whereas anxiety may be diagnosed if there is avoidance of fearful situations, apathy has been defined as an “action-avoidance” syndrome (Skidmore et al., 2013), with both of these deleteriously affecting goal-directed behaviour.

Indeed, it may not be meaningful to attempt to separate the concepts of apathy and mood disorder in PD. Commonalities across apathy, depression, and anxiety may reflect the disruption of serotonergic pathways known to accompany dopaminergic denervation in PD (Maillet et al., 2016; Thobois et al., 2017). These disease-general abnormalities may then interact with patient-specific factors, such as response to disability and isolation, to help shape the expression of these non-motor symptoms. Similarly, Simpson et al. (2015) found that patients with PD and apathy described this as a response to the consequences of impairment, related to low mood and hopelessness. This suggests that attempts to isolate a pure apathy syndrome to the exclusion of depression and/or anxiety may be ignoring the patient’s private experience.

The second aim of this study was to determine the cognitive profile of apathy in PD. Previous studies have varied in their inclusion of patients with cognitive impairment and in their assessment of cognitive function, with some relying upon screening tools only. Our extensive neuropsychological assessment revealed that PD with pure mood disorder is associated with a reduction in non-verbal intellectual functioning, as indexed by PIQ. Although our sample sizes of further subgroups were small, we did not find any evidence of additional losses in cognitive function that were present in patients with either additional apathy or pure apathy. Correlational analyses confirmed that self-ratings of apathy were not associated with a general level of intellectual functioning, but rather explained by mood disorder, mostly level of depression. These findings are consistent with neuroimaging studies, which have revealed anatomical changes within the limbic system shared across presentations of apathy, depression, and anxiety in PD (Wen et al., 2016; Thobois et al., 2017).

These findings may be important for the treatment of apathy. Rather than attempting to treat apathy as an isolated symptom, it may be more useful to consider it as part of a suite of conjoined neuropsychiatric symptoms that would likely benefit from treatments currently designed for depression and anxiety. In addition to medical therapies, this may also include psychological interventions. Indeed, a recent study reported significant reductions in apathy alongside symptoms of anxiety and depression in a group of patients with PD following a course of cognitive behavioural therapy (Berardelli et al., 2018).

Of course, there are a number of limitations to our study. First, our study was retrospective in design, limiting interpretation of some findings. Second, we have focussed exclusively on self-ratings of apathy. There are low levels of agreement between patient and carer ratings of apathy (McKinlay et al., 2008), perhaps betraying intrusion of rater factors, such as level of carer burden (Schiehser et al., 2013). Although self-ratings of apathy have been found to be highest in PD (Radakovic et al., 2018; Valentino et al., 2018), it may still be possible that in some cases, patients demonstrate insufficient insight into their behaviour. Therefore, future studies should include multiple measures of apathy, perhaps including both self- and proxy-ratings. Third, our study consisted of unequal subgroup sizes. However, the extremely small prevalence rate of “pure apathy” suggests that it may be difficult to achieve sufficiently large and equal-sized subgroups in this condition. Fourth, we did not consider potential apathy subtypes. Previous research has found that apathy in PD may reflect emotional-affective and/or auto-activity subtypes, rather than a cognitive subtype (Pagonabarraga et al., 2015), and our findings of an overlap between apathy and mood disorder supports this. Future studies wishing to investigate this further may choose to adopt a dimensional scale of apathy, such as the Dimensional Apathy Scale (Radakovic and Abrahams, 2014), but with the caveat that this tool is less able to detect apathy in PD (Santangelo et al., 2017; Mele et al., 2019). Fifth, we did not also include years of education in our analyses. However, there were no significant differences in NART score across subgroups, suggesting that that education level was not related to the manifestation of apathy in our sample. Finally, our study did not consider PD motor symptoms or stage. It has been suggested that the type and/or severity of apathy may be associated with particular motor symptoms (e.g., Ziropadja et al., 2012) and/or evolve with progression of the disease (Maillet et al., 2016). However, we note that in our study, there were no significant differences in disease duration across subgroups, and our previous research has shown no relation between motor scores and apathy (Foley et al., 2017).

In sum, we have shown that pure apathy is very rare in PD; when apathy is present in PD, it overlaps greatly with both depression and anxiety, suggesting that apathy in PD may be in large an epiphenomenon of mood disorder, presenting as part of a behaviour non-motor triad (Maillet et al., 2016). These findings are supported by our cognitive assessment, which has shown that cognitive functioning in PD is lower in patients with mood disorder, but without any additional deficits caused by comorbid apathy.

## Data Availability Statement

The data analysed in this study are subject to the following licenses/restrictions: The data are not publicly available due to privacy or ethical restrictions. Requests to access these datasets should be directed to LC, Lisa.Cipolotti@nhs.net.

## Ethics Statement

The studies involving human participants were reviewed and approved by the Institute of Neurology Joint Research Ethics Committee UCLH. Written informed consent for participation was not required for this study in accordance with the national legislation and the institutional requirements.

## Author Contributions

JF conceived, organised, and executed the study, with guidance, review, and critique from LC. Both authors contributed to the writing of the manuscript.

## Conflict of Interest

The authors declare that the research was conducted in the absence of any commercial or financial relationships that could be construed as a potential conflict of interest.

## Publisher’s Note

All claims expressed in this article are solely those of the authors and do not necessarily represent those of their affiliated organizations, or those of the publisher, the editors and the reviewers. Any product that may be evaluated in this article, or claim that may be made by its manufacturer, is not guaranteed or endorsed by the publisher.
